# Valosin-Containing Protein (VCP): A Review of Its Diverse Molecular Functions and Clinical Phenotypes

**DOI:** 10.3390/ijms25115633

**Published:** 2024-05-22

**Authors:** Carly S. Pontifex, Mashiat Zaman, Roberto D. Fanganiello, Timothy E. Shutt, Gerald Pfeffer

**Affiliations:** 1 Hotchkiss Brain Institute, Department of Clinical Neurosciences, Cumming School of Medicine, University of Calgary, Calgary, AB T2N 1N4, Canada; cspontif@ucalgary.ca (C.S.P.); mashiat.zaman1@ucalgary.ca (M.Z.); timothy.shutt@ucalgary.ca (T.E.S.); 2 Alberta Child Health Research Institute, Department of Medical Genetics, Cumming School of Medicine, University of Calgary, Calgary, AB T2N 1N4, Canada; 3 Department of Biochemistry and Molecular Biology, University of Calgary, Calgary, AB T2N 1N4, Canada; 4 Faculty of Science and Engineering, Université Laval, Quebec City, QC G1V 0A6, Canada; robertofanganiello@gmail.com; 5 Heritage Medical Research Building 155, 3330 Hospital Dr NW, Calgary, AB T2N 4N1, Canada

**Keywords:** valosin-containing protein, multisystem proteinopathy, genetic diagnosis, genotype–phenotype correlation, autophagy, mitophagy, lysophagy, stress granules, ERAD, cell cycle control, DNA damage response, apoptosis, SUMOylation, ubiquitination, skeletal muscle, osteoclast, neuron

## Abstract

In this review we examine the functionally diverse ATPase associated with various cellular activities (AAA-ATPase), valosin-containing protein (VCP/p97), its molecular functions, the mutational landscape of VCP and the phenotypic manifestation of VCP disease. VCP is crucial to a multitude of cellular functions including protein quality control, endoplasmic reticulum-associated degradation (ERAD), autophagy, mitophagy, lysophagy, stress granule formation and clearance, DNA replication and mitosis, DNA damage response including nucleotide excision repair, ATM- and ATR-mediated damage response, homologous repair and non-homologous end joining. VCP variants cause multisystem proteinopathy, and pathology can arise in several tissue types such as skeletal muscle, bone, brain, motor neurons, sensory neurons and possibly cardiac muscle, with the disease course being challenging to predict.

## 1. Introduction

Valosin-containing protein (VCP/p97) belongs to the AAA+ (ATPases associated with diverse cellular activities) family of chaperone-like proteins and has a wide array of pleiotropic functions within the cell. Among them are the mediation of ubiquitin-dependent cellular processes through the ubiquitin–proteasome system (UPS) [1], protein quality control [2,3], transcription factor processing [4], membrane fusion [5], cell cycle control [6] and the regulation of autophagy [7]. VCP’s expression is ubiquitous and abundant, representing around 1% of the cytoplasmic protein content [8]. It is also highly conserved in all organisms belonging to the Eukarya domain, and is indispensable for mammalian development [9]. Pathogenic variants in the gene encoding VCP cause multisystem proteinopathies (MSPs), a set of genetic diseases characterized by myopathy, bone disease and/or neurodegeneration of central and/or peripheral nervous systems [10].

Multisystem proteinopathy 1 (MSP1), also known as IBMPFD (inclusion body myopathy associated with Paget’s disease of bone and frontotemporal dementia), or VCP-disease [10,11] has been associated with more than 100 heterozygous missense pathogenic variants in *VCP*. Other MSP genes include heterogeneous ribonucleoprotein A2B1 (*HNRNPA2B1*), heterogeneous ribonucleoprotein A1 (*HNRNPA1*), Matrin3 (*MATR3*), sequestosome-1 (*SQSTM1*) and optineurin (*OPTN*) [11]. MSP1 is a progressive disorder of autosomal dominant inheritance, and its main pathological hallmarks are twofold: (1) the presence of ubiquitin-positive protein aggregates in muscle tissues of patients affected by myopathy [12] and (2) intranuclear TDP-43+ inclusions in frontotemporal dementia [13]. In Paget’s disease of bone (PDB), abnormally large osteoclasts containing nuclear inclusions resemble the muscle pathology findings [14]. Additional clinical presentations have been rarely described in association with pathogenic variants in *VCP* which are distinct from the classic MSP phenotypes. Clinical management for these conditions has benefited from the development of consensus guidelines [12,15].

Additional phenotypes beyond MSP have been described in patients harboring pathogenic variants in VCP. Individual rare cases of *VCP* mutations have been associated with single system phenotypes such as Charcot-Marie-Tooth Disease type 2Y [16]. Recently, VCP pathogenic variants have also been associated with a childhood-onset neurodevelopmental disorder, characterized by intellectual and developmental disability, hypotonia and macrocephaly. These variants are distinguished by a reduction in in vitro ATPase activity and are variants which have never been associated with adult-onset presentations such as MSP [17]. There are also descriptions of *VCP* variants associated with Parkinson’s disease [18,19]. Complex phenotypes may occur in the presence of secondary genetic diagnoses, such as a recently described case with a coincident intermediate expansion in *SCA2* [20]. However, the vast majority of reported cases of VCP-linked disease are associated with MSP phenotypes, the majority of which are associated with two variants at presumed mutational hot spots (residues 155 and 159), and it is likely that atypical phenotypes are underrecognized, misdiagnosed or not yet characterized [21].

Overall, the pathologic findings of MSP1 suggest that abnormal protein clearance is the major mechanism by which *VCP* variants cause human disease. In MSP caused by mutations in genes other than *VCP* (recently reviewed) [11], it has also been suggested that the common molecular mechanism relates to disruption of two major protein clearance pathways: the UPS and autophagy [22]. Based on this information, our discussion of molecular mechanisms is primarily focused on the roles of VCP in protein quality control, although we include some discussion of other mechanisms that are affected by mutations associated with human disease. In this review paper we outline the ever-expanding role of VCP and its variants in the cell, including its roles in protein quality control, autophagy, lysophagy, mitophagy, pexophagy, granulophagy, ERAD, the mitochondria, DNA replication and mitosis, DNA damage response and cell death.

## 2. Structure and Function of the VCP Protein

VCP forms a hexameric double-ring structure and has the following different functional domains: the N-terminal ubiquitin-binding domain, the D1 and D2 ATPase domains, two linker domains (L1 and L2) and a carboxyl-terminal domain (C-domain) [23]. The L1 and L2 domains overlap and form a central pore, and the presence of Zn^2+^ ions in the central pore indicate that interaction with substrate occurs on the protein’s exterior [23]; cryo-electron microscopy (CryoEM) and crystal structures indicate that the N-terminal domain interacts with cofactors and recognizes ubiquitin-tagged proteins. The diverse functional nature of VCP may be attributed to its many associated cofactors, which allows VCP to interact with multiple surfaces, structures and organelles within the cell [1]. The ubiquitinated target proteins are then pulled through the D1 and D2 ATPase domains via interactions with hydrophobic residues inside the central pore, thus engaging and unraveling proteins through the pore so that they may be degraded by the proteasome [24]. Structural analyses illustrate that the N-terminal domain has two structural conformations, an inactive closed coplanar conformation, or an active and open flexible conformation [25]. Intriguingly, most of the pathogenic mutations of *VCP* occur at the interface between the N-domain and the D1 domain, with some variants appearing to become fixed in the open conformation and sometimes exhibiting higher ATPase activity [25]. VCP is also regulated by post-translational modifications and is phosphorylated and acetylated at the C-terminus [26], while lysine residues (K60, K62, K63, K136 and K164) at the N-terminus are SUMOylated after exposure to oxidative stress or proteasome inhibition [27]. After wild-type VCP is exposed to stressors, there is a significant increase in the localization of VCP to stress granules and nuclei [27]. A number of disease-causing MSP *VCP* variants (including R95G, G97E, R155C, R159H, R191Q and A232E) have reduced SUMOylation in response to stressors, and investigators observed that this has led to reduced hexamer assembly, altered cofactor binding and the reduced localization of VCP to stress granules and nuclei [27].

### Pathological Mechanism: Dominant Negative or Toxic Gain-of-Function?

The pathological mechanism of MSP *VCP* variants is presumed to be related to a dominant negative effect [28,29] or a potentially toxic gain-of-function [30]. With the vast number of disease-causing variants [21], there may be cases where the principal dysfunction is due to a dominant negative effect, a toxic gain-of-function effect or, in some cases, both might be contributing simultaneously to different degrees and may be altered depending on the variant. There is a strong case for a dominant negative effect, where it is likely that heterozygous variants impact the hexameric ring structure. The hexameric VCP ring will have some functional monomers and some dysfunctional monomers whose mutations may lead to conformational changes. This mixture of monomers compromises the binding of adaptors and cofactors (by either increasing or decreasing affinities) and thus selectively impairing the recruitment and unwinding of some ubiquitinated targets. This example may be the case when variant VCP fails to migrate to certain organelles, like mitochondria [31], or when VCP fails to translate to stress granules [27]. In another case, Weihl et al. found that, although one of the most common and well-studied variants, R155H, has normal ATPase activity, there was an impairment of endoplasmic reticulum-associated degradation (ERAD), described in subsequent sections [32]. Arhzaouy et al. found that knocking out *VCP* in mouse skeletal muscle caused substantial necrosis and the accumulation of autophagic proteins, noting that the heterozygous knock-in of R155H in mouse skeletal muscle had a comparatively milder phenotype [33]. However, Cheng et al. (2022) make the case for the toxic gain-of-function hyperactivity model of VCP, using a knock-in mouse model of R155H *VCP* in conjunction with the D2 domain-targeted VCP inhibitor CB-5083; investigators noted an improvement in muscle pathology in the knock-in variants in the presence of the inhibitor [34]. It would be interesting to test this hypothesis in a well-established hyperactive variants such as A232E VCP, to see if the impact of VCP inhibitors is even more prominent [35,36], while keeping in mind that different variants could be differentially sensitive to VCP inhibitors [35]. There may also be the impaired binding of cofactors and adapters, in addition to changes in ATPase activity. It is possible that there may be some ubiquitinated VCP targets that fail to recruit to VCP and therefore accumulate in the case of dominant negative effects. At the same time, other VCP targets may be disproportionately targeted and degraded in the case of toxic gain-of-function, both of which could lead to cellular dysfunction. In studying the enzyme kinetics of A232E, R155H and T262A, there was an apparent decrease in the dissociation constant with ATP as substrate, suggesting that variants had a higher affinity for ATP and left the hexameric structure of VCP in the open conformation, increasing the binding of the VCP adapters nuclear protein localization protein 4 homolog (*Npl4*/NPLOC4) and ubiquitin fusion degradation protein 1 homolog (UFD1L), which increased substrate processing [37].

When VCP was knocked out in mouse muscle, pathogenicity was imminent, which suggests that the loss-of-function mutation of VCP can cause disease. However, the heterozygous knock-in of pathogenic VCP was also pathogenic, with a milder phenotype than a complete knock-out model. With VCP’s hexameric protein structure, it is reasonable to assume that the pathogenic variant likely interferes with wild-type function in heterozygous systems, which implies that the mutation might be classified as dominant negative. Supporting this notion is the evidence that demonstrates that SUMOylation and subsequent cofactor and adapter binding are altered in pathogenic variants. Some, but not all, pathogenic variants exhibit hyperactivity indicating toxic gain-of-function effects. We propose that MSP VCP alterations might be both dominant negative and toxic gain-of-function, and different variants have subtle variable cellular effects. Each variant may require careful scrutiny to fully understand how the mutations are influencing VCP’s behavior at the level of the hexamer, in order to best predict the response to future treatment.

## 3. Role of VCP in the Cytosol

**Figure 1:**
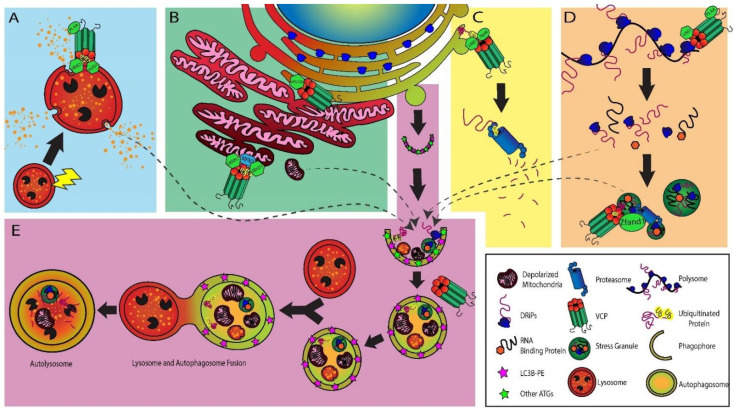
**Diverse functions of cytosolic VCP in the proteasome and autophagy systems.** (**A**) Lysophagy: VCP responds to lysosomal damage with cofactors PLAA, UBXD1 and YOD1, preparing the lysosome for recruitment to the phagophore. (**B**) Mitophagy: VCP is recruited to depolarized mitochondria with UBXN1 and UBXD1 cofactors to extract MFN2 to facilitate fragmentation of depolarized mitochondria in preparation for mitophagy, and VCP interacts with VPS13D to regulate the tethering of the ER to mitochondria. (**C**) ERAD: VCP and cofactors UFD1 and NPL4 extract misfolded proteins from the ER, extracted proteins are degraded by the proteasome. (**D**) Stress granules: VCP cofactors UFD1 and PLAA degrade defective ribosomal products during polysome disassembly, which plays a role in stress granule assembly. ZFAND1 recruits VCP and 26S proteasome to stress granules for their clearance by autophagy. (**E**) Autophagic flux: The phagophore buds off from the omegasome of the ER and defective proteins and organelles are recruited to the growing phagophore. The phagophore closes around the cellular waste as ATG proteins are recruited and removed from the membrane. The mature autophagosome fuses with the lysosome, forming the autolysosome where contents are degraded. VCP may be implicated in autophagosome maturation, although the precise mechanisms have yet to be elucidated. The arrows from panels A, B and D indicate that dysfunctional proteins, organelles and stress granules are recruited to the growing autophagosome.

The protein quality control system of the cell is regulated by two interdependent cellular processes: the ubiquitin–proteasome system (UPS) and macroautophagy, also referred to as autophagy [38]. The UPS system degrades small soluble proteins and aggregates, while the autophagy system manages the degradation of large aggregates and organelles [38]. VCP is important in both processes. VCP is involved in regulating translation, cooperates with the proteasomal system by unraveling ubiquitinated proteins prior to proteasomal degradation, and plays a critical role in preparing organelles and larger aggregates for autophagic degradation. A discussion of VCP’s various functions is provided below and summarized in Figure 1.

### 3.1. Autophagy

After the phagophore buds off from the omegasome, cellular waste, such as bacteria, viral particles, protein aggregates or damaged and fragmented organelles, are loaded into the inner membrane of the expanding phagophore [39]. Subsequently, the phagophore closes, forming the waste-carrying vesicle, the autophagosome. Before the autophagosome can fuse with the lysosome to degrade waste products, the autophagosome must undergo a maturation process, whereby ATG proteins must be removed from the membrane to facilitate the docking of the autophagosome–lysosome fusion machinery [39]. The maturation of the autophagosome is complete when the autophagosome fuses with the lysosome.

In mouse muscle knock-out (KO) experiments, either VCP or autophagy-related 5 (ATG5) KO experiments were compared. ATG5 is a protein that is essential to the expansion of the phagophore; thus, an ATG5 KO would inhibit the formation of the autophagosome and autophagy [33]. It was observed that, when ATG5 was knocked out, muscle pathology was normal, but when VCP was knocked out, there were abnormalities typical of MSP myopathies including rimmed vacuoles [33]. These results suggest that, although VCP plays a prominent role in autophagy, with numerous autophagy defects in MSP, early autophagy inhibition alone does not recapitulate the MSP phenotype, lending support to the idea of a toxic gain-of-function model of VCP [33]. The drug colchicine, commonly used to treat gout, can induce vacuolar myopathy after long-term medical administration [40,41,42]. Its mechanism of action activates autophagy but also depolarizes microtubules which secondarily prohibits autophagosome and autolysosome fusion [43]. We ask if it is possible that part of the pathological mechanism, then, can be attributed to the accumulation of defective autophagic bodies, either autophagosomes or lysosomes.

It is important to consider how the standards for assessing autophagy and autophagic flux have changed since early publications on VCP and autophagy dating back to 2009, as studies using different approaches have reported conflicting results. Ju et al. determined that, while studying VCP mutants R155H, A232E and ATPase-inactive E578Q, that autophagosome maturation was impaired. Meanwhile Tress et al. concludes that, in basal autophagy, but not in serum-starved conditions, knocking down VCP or the overexpression of R155H, A323E or dominant negative variants impaired the maturation of the autophagosome [44]. In 2016, Bayraktar et al. found an increase in autophagosomes and autolysosomes in a P137L VCP mutant across several cell types [45]. R155C and R191Q variants are aggregate prone, while P137L variants stimulate autophagosome and autolysosome formation [46].

However, these conclusions were based on results from tandem EGFP-mCherry-LC3B experiments, which have technical limitations. For example, EGFP retains some mild fluorescence at a lysosomal pH, and a neutral pH buffer like the common fixative agent paraformaldehyde can restore GFP fluorescence that has been reduced due to lowered pH conditions [47,48,49,50]. Published guidelines exist for the standardization of methodology in studies of autophagy, with discussion of mitigation strategies [51]. Experiments that use multiple transfected proteins and overexpression also carry important limitations [52]. In the specific case of VCP, the overexpression of mutant VCP leads to distorted ratios of healthy versus mutant subunits in VCP hexamers, which risks further imposing deficits not observed in endogenous systems. Thus, caution should be taken in interpreting such results.

### 3.2. Lysosomes

Much of the discussion so far has been on the impact of VCP in muscle, but the second most common manifestation of MSP is PDB. Both skeletal muscle and osteoclasts in bone are multinucleated cells [53], and osteoclasts maintain and repair bone by secreting bone-dissolving acids from secretory lysosomes. PDB patients have more osteoclasts, with a greater number of nuclei per osteoclast, suggesting excessive osteoclast fusion. In PDB patients, osteoblasts are overcompensating for the bone destruction of the osteoclasts by upregulating repair pathways, leading to abnormal bone formation [53,54]. The secretion of enzymes and acids responsible for bone resorption is mediated by secretory lysosomes, which fuse with the ruffled border of bone [55]. One possible pathogenic mechanism we suggest for PDB in VCP-MSP might be that osteoclasts have excessive intracellular accumulation and secretion of lysosomes, whereby osteoblasts are forced to overcompensate for bone destruction.

The fusion of the autophagosome and the lysosome represents the final stage of autophagy, whereby waste products are degraded in the highly acidic compartment of the lysosome. Should the membrane of the lysosome become permeabilized or ruptured beyond repair, the lysosome may be subjected to selective autophagy of the lysosome, termed lysophagy [56]. Recent insights have found that VCP regulates lysosomal integrity after lysosomal or endolysosomal damage alongside the ubiquitin-conjugating enzyme and VCP cofactor, ubiquitin-conjugating enzyme E2 Q family-like (UBE2QL1) [56,57,58]. Upon lysosomal damage, UBE2QL1 appears to play a role in ubiquitinating K48 and K63 chains, followed by the recruitment of autophagic receptors sequestosome1 (SQSTM1), tax1 binding protein 1 (TAX1BP1), optineurin (OPTN) and calcium-binding and coiled-coil domain-containing protein 2 (CALCOCO2/NDP52) [56]. In addition, VCP is known for extracting K48-ubiquitinated proteins for proteasomal degradation [57]; VCP is also recruited to ubiquitinated lysosomes as a part of the ELDR complex, which consists of YOD1 deubiquitinase (YOD1), UBX domain protein 6 (UBXD1/UBXN6), phospholipase A-2-activating protein (PLAA) and VCP [59]. The VCP variants R155H, L198W, A232E and E578Q all had indications of impaired clearance of damaged lysosomes [59]. When VCP was knocked out in the skeletal muscle of adult mice, damaged lysosomes, necrosis and the accumulation of autophagic proteins were observed [33].

### 3.3. Mitochondria

**Figure 2:**
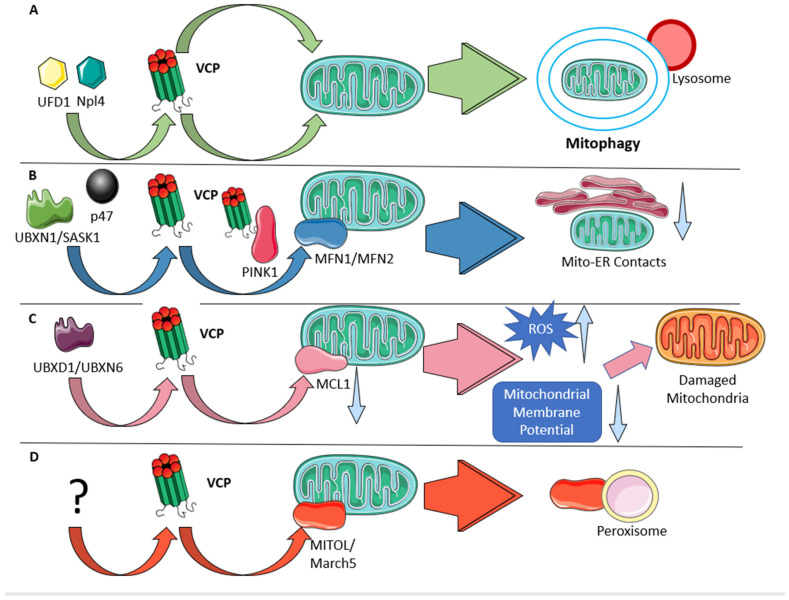
**The interactions of VCP with mitochondrial proteins.** The figure represents the functions of VCP associated with mitochondria, with arrows indicating interactions between proteins. (**A**) UFD1 and NPL4 first complex with VCP and before being recruited to a multitude of outer mitochondrial membrane proteins which are extracted for proteasomal degradation in the cytosol while also regulating mitophagy. (**B**) VCP cofactors UBXN1/SASK1 and p47 bind to VCP. This complex is associated with degradation of MFN1/MFN2, which is mediated through contact with PINK1, andh leads to a decrease in mitochondria–ER contact sites. (**C**) VCP binds with UBXD1/UBXN6 leading to degradation of MCL1, causing loss of mitochondrial membrane potential. (**D**) VCP can target the MITOL/March5, which leads to detachment from the mitochondrial outer membrane and association with peroxisomes. The question mark indicates that we currently do not know which VCP cofactors/adaptors bind to VCP to facilitate this interaction.

VCP-associated MSP patients have altered mitochondrial bioenergetics, including decreased ATP, decreased spare respiratory capacity and increased mitochondrial enzyme proteins, indicating that VCP may play some role in mitochondrial integrity (R155H and R155S) [60]. VCP is involved in the quality control of outer mitochondrial membrane (OMM) proteins and is thus important for helping to maintain mitochondrial function, mitochondrial dynamics and mitophagy. VCP is recruited to the OMM, where it facilitates the extraction of several integral OMM proteins for their subsequent proteasomal degradation [61,62,63]. Some of the more well-characterized VCP OMM target proteins identified to date include the mitochondrial fusion proteins MFN1 [61,62] and MFN2 [62], the E3 ubiquitin ligase membrane-associated ring finger (C3HC4) 5 (MITOL/March5) [64], the apoptosis regulators myeloid cell leukemia 1 (MCL1) [61,65], Bcl-2 related ovarian killer (BOK) [66] and solute carrier family 25 member 46 (SLC25A46), a protein linked to hereditary motor and sensory neuropathy and pontocerebellar hypoplasia [67]. Important protein cofactors/adaptors that help mediate VCP function on the OMM include UBXD1/UBXN6, which mediates valerophenone synthase (VPS) recruitment to mitochondria [68], as well as VCP cofactors ubiquitin recognition factor in ER-associated degradation 1 (UFD1) [69], Npl4 [31,69], neutrophil cytosol factor 1 (p47) [31] and UBX domain protein 1 (UBXN1/SAKS1) [70], which are recruited to mitochondria along with VCP. When UBXN1 is diminished, VCP translocation to mitochondria is impaired [70] (Figure 2). Consistent with a gain-of-function effect, pathogenic VCP variants R95G, R155H and E305A bind more efficiently to UFD1, Npl4 and p47 cofactors [71], and variants R95G, R155C, R155H, R155P, A232E and T761E all exhibit accelerated cellular protein aggregation [72]. Moreover, the exogenous expression of the pathogenic VCP variants R155H, R191Q and A232E impairs the mitochondrial recruitment of VCP and its adaptor proteins in mammalian cells [31]. The role of VCP in mediating mitochondrial function is supported by the fact that pathogenic VCP variants lead to mitochondrial dysfunction in patient cell lines [60,73] and mouse disease models [60,74], as well as by work in both yeast [75,76] and Drosophila models [30,31,69,77,78]. Due to its role in OMM protein extraction, VCP is implicated into several interrelated mitochondrial functions that ultimately impact the role of mitochondria in energy production, including mitochondrial fusion, mitophagy, mitochondria–ER contacts (MERCs), mitochondrial protein import and mitochondrial motility. Some of these functions are summarized in Figure 2.

Before depolarized or damaged mitochondria can be loaded into the autophagosome, mitochondria must undergo fission into small enough fragments for mitochondrial autophagy (mitophagy). In this regard, one of the most thoroughly characterized OMM VCP targets is the multifunctional protein MFN2, which is a mitochondrial fusion protein that is also implicated in mitophagy. Mutations in *MFN2* cause the peripheral neuropathy Charcot-Marie-Tooth disease type 2A (CMT2A) [79], with a highly variable axonal-predominant phenotype [80]. Interestingly, in a small number of cases, CMT2 phenotypes have been linked to VCP variants G97E and E185K [16,81]. In addition, to its well-recognized roles in mitochondrial fusion and mitophagy [82], MFN2 is also involved in MERCs [83] and mitochondrial motility [84], and is thus a promising candidate to mediate the effects of VCP on mitochondrial function. With respect to the related roles of MFN2 and VCP in mitophagy, after mitochondria become depolarized, PINK1 becomes stabilized allowing the recruitment of Parkin to the damaged mitochondria [85]. Activated Parkin ubiquitinates MFN2, recruiting VCP, which extracts MFN2 followed by mitochondrial fragmentation and loading into the autophagosome [68,70]. Additionally, VCP interacts directly with PINK1, which regulates the phosphorylation of p47 [86]. Thus, through this role in mitophagy, VCP is important for mitochondrial quality control beyond just the removal of OMM proteins. Further linking VCP to another neurodegenerative disease, it is also notable that PINK1 and Parkin are encoded by Parkinson’s disease genes [69,87].

Of the mitochondrial functions downstream of VCP, MERCs are relevant to neurodegenerative diseases in general [88], and to ALS specifically [89,90]. The VCP-mediated removal of MFN2 reduces MERCs [91], which is proposed to facilitate the further recruitment of Parkin and the ubiquitination of OMM proteins to stimulate robust mitophagy [92]. Notably, VCP is also involved in the extraction of the ER-resident protein VAPB, another key protein that mediates MERCs [93] and is also linked to ALS [89]. Meanwhile, MERCs are also impaired by repeat expansions in C9orf72, the most common cause of familial ALS [90]. Thus, it seems likely that downstream impairments to MERCs are likely to contribute to the pathology of pathogenic *VCP* variants.

Beyond mitophagy and MERCs, VCP is also implicated in mitochondrial protein import. The vast majority of the ~1000 mitochondrial proteins are encoded in the nucleus, translated in the cytosol and imported into mitochondria through a dedicated protein import machinery that can be coupled to translation. In yeast, the VCP homolog Cdc48p removes partially imported proteins that are arrested in the mitochondrial protein import channel [94]. Notably, also in yeast, the VCP-associated protein Vms1p interacts with ribosomes at the mitochondrial surface to restrict the accumulation of aggregation-prone proteins [95]. It remains unknown whether Cdc48p also impacts the role of Vms1p in these mitochondria-associated ribosomes, or whether VCP also impacts mitochondrial protein import in mammalian cells.

Drosophila models have been especially valuable in understanding how VCP impacts mitochondrial functions and the role it plays in disease. Downregulation of Drosophila VCP (dVCP) reduces the mitochondrial density in axons due to enhanced mitochondrial retrograde transport, a phenotype that is recapitulated by the introduction of pathogenic VCP variants R152H and A229E into dVCP [96]. Relevant to myopathy, when studying the effect of dVCP in Drosophila muscle, the loss of dVCP led to the increased expression of the MFN2 homolog mitochondrial assembly regulatory factor (MARF) and hyperfused mitochondrial networks, while dVCP overexpression had the opposite effects [30]. Moreover, dVCP overexpression was able to suppress mitochondrial defects in flies lacking PINK1 or Parkin [69]. With respect to pathogenic variants, the exogenous expression of dVCP harboring the R152H or A229E variants (corresponding to human VCP R155H and A232E), led to defective mitochondria, excessive cell death and muscle loss [77]. Expression of these variants led to the excessive downregulation of MARF, indicating that they were hyperactive. Further supporting the notion that the R152H and A229E variants are hyperactive, their overexpression could also rescue mitochondrial defects in Parkin-null flies [69]. Remarkably, VCP inhibitors blocked mitochondrial defects and muscle tissue damage in dVCP variant flies and patient fibroblasts, suggesting their potential for therapeutic use [30]. Meanwhile, the overexpression of MARF could restore the fragment mitochondrial morphology induced by overexpressing dVCP, but was unable to rescue the cell death or muscle loss, suggesting that, while mitochondrial dysfunction likely contributes to the disease pathology, it is not the only downstream consequence of VCP dysfunction that is involved [30].

Mouse knock-in models of VCP variants also act as valuable disease models. One of the first studies of mice overexpressing human VCP with the R155H or A232E variants faithfully recapitulated IBMPFD phenotypes and showed abnormal mitochondria within the muscle niche [97]. Similarly, heterozygous VCP R155H knock-in mice have mitochondrial dysfunction [98] and showed progressive muscle, bone, brain and spinal cord phenotypes [99]. Meanwhile, homozygous VCP R155H knock-in mice have very short life spans (2–3 weeks) and exhibit myopathy and distinct mitochondrial phenotypes, including increased lipid composition, increased oxidative phosphorylation and elevated NADH production [74]. Hence, the phenotypes associated with VCP dysfunction and the particular hallmark changes seen in mitochondria can be recapitulated in mouse models and suggests that disruptions to mitochondrial function is a significant consequence of *VCP* variants.

### 3.4. Stress Granule Formation and Clearance

Stress granules are non-membrane-bound cytoplasmic complexes composed primarily of RNA-binding proteins, RNA, ribosomal machinery and translation initiation factors [100,101]. Dysfunctional stress granules have been implicated in many neurodegenerative and neuromuscular diseases, including Alzheimer’s disease, amyotrophic lateral sclerosis (ALS) and myopathy [102,103,104,105].

Functionally, stress granules selectively inhibit translation in response to starvation, oxidative stress, viral infection, DNA damage, proteasome inhibition, endoplasmic reticulum (ER) stress, hyperosmotic stress, heat shock and cold shock [100,101]. VCP’s role in the management of cellular waste extends beyond basal waste management and includes acute and long-term responses to cellular stress. It has been observed that, when VCP is knocked down by siRNA, stress granule formation is impaired and stress granules appear to retain more defective ribosomal products, suggesting VCP’s role in removing defective products from stress granules [106]. Moreover, VCP cofactors UFD1L (n-terminal interacting) and PLAA (c-terminal interacting), when depleted, alter the formation of stress granules and morphology depending on cellular stress, as they appear to play a role in degrading defective ribosomal products [106]. Autophagy initiators ULK1/2 phosphorylate VCP to stimulate stress granule disassembly [107]. SUMOylation of VCP enables VCP to translocate to stress granules and many *VCP* pathogenic variants have a reduction in SUMOylation [27]. Another study indicated that VCP is recruited to stress granules by zinc finger AN1-type-containing 1 (ZFAND1) after exposure to the oxidative stressor sodium arsenite [108]. HeLa cells transfected with VCP-R155H variants have 10% more stress granules 2 h after a stressor compared to wild-type cells, and, when ZFAND1 was knocked down in conjunction with either a VCP R155H variant or with wild-type cells, 40% of cells retained stress granules after 2 h [108]. This finding indicates that ZFAND1 supports arsenite-induced stress granule clearance. Furthermore, this group was able to demonstrate that ZFAND1 specifically supports stress granule degradation via the proteasome, and, when ZFAND1 was depleted, stress granules were instead cleared away by the autophagy system [108].

When the proteasomal system of a cell is overwhelmed, the autophagic systems can compensate when the cell undergoes large-scale protein misfolding. Importantly, the autophagy system is also significantly impaired in VCP mutants, as discussed at length in subsequent sections. Managing the clearance of stress granules is relevant because the persistence of cellular stress granules has been linked to the pathology of myopathy and neurodegenerative diseases. For instance, a pathogenic variant of the stress granule protein T cell intracellular antigen-1 (*TIA1*) gene can cause Welander distal myopathy [109]. A separate variant with a low population frequency is not pathogenic on its own but can modify the phenotype of *SQSTM1* variants to present as a distal myopathy [110,111] and is identified in a disproportionate number of distal myopathy cases [110]. Other stress granule proteins, FUS and TDP-43, are well-established causes of ALS/FTD; notably, several VCP variants (R159C, N387T, R662C) have so far been identified as causing 1–2% of sporadic ALS cases [112]. Exactly how changes in stress granule dynamics contribute to pathology is not fully understood. We hypothesize that, perhaps when proteins related to translation or other cellular functions are trapped within the stress granules for extended periods, it prevents the restoration of translation homeostasis or that stress granule aggregates are additively dysfunctional as they contribute to overwhelming the autophagy systems.

### 3.5. Endoplasmic Reticulum

VCP is involved in ER processing and ER-associated degradation (ERAD) and also mediates the biogenesis of the ER [5,6]. After translation, one third of nascent proteins are transported into the ER, where they undergo appropriate folding and maturation and, in cases where proteins fail to assemble or fold, these proteins must be degraded, which involves VCP [113,114]. VCP is also a regulator of ER stress [115], as VCP complexes with the prominently studied adapters Ufd1 (ubiquitin fusion degradation 1) and Npl4 (nuclear protein localization 4) form a complex to extract and unwind misfolded proteins from the ER surface before transfer to the proteasome [2,116,117,118,119]. The inhibition of VCP leads to significant ER stress, cell cycle arrest and apoptosis [120]. Beyond this, VCP mediates ER–mitochondria interactions via VPS13D, a protein involved with mitochondrial clearance by mitophagy, and in the regulation of mitochondrial fission [93]. The R155H mutation has normal ATPase activity, but impairment of ERAD [32].

Furthermore, the ER region called the omegasome is the nucleation site of the proto-autophagosome structure, the phagophore, which suggests the possibility that impairments in VCP through ER stress may influence even the earliest stages of autophagy, though this has not been studied. In this way, VCP may act as an additional stressor to the autophagy and protein clearance systems by impairing misfolded protein clearance within the ER.

## 4. VCP and Autophagy-Dependent Cell Death

The precise role that autophagy plays in cell death is difficult to characterize, as autophagy may play different roles in cell death depending on the physiological context. For instance, autophagy-mediated cell death takes place when autophagy is a precursor to apoptosis and to subsequent cell death, while autophagy-dependent cell death occurs in the absence of apoptosis or necroptosis. In autophagy-associated cell death, autophagy occurs alongside apoptosis, but apoptosis is the primary cause of cell death. There is also the possibility that apoptosis and autophagy simultaneously induce cell death, or that apoptosis may lead to both autophagy and cell death at once [121]. To complicate matters, the mechanism may vary greatly by organism, cell type and context [121]. That being said, there appears to be a link between autophagy and the formation of multinucleated terminally differentiated myotubes, whereby autophagy prevents apoptosis [122]. The observation that prolonged autophagy can contribute to cell death in hippocampal neural stem cells (HNC) led to further works that suggest an emerging role for VCP in autophagic cell death and apoptosis [123]. In the presence of insulin, VCP facilitates basal autophagy through the maturation of the autophagosome HNCs. However, upon insulin withdrawal, VCP regulates the initiation of signaling between autophagy, autophagic cell death and apoptosis [124]. Short-term and long-term ablation of the autophagy-essential E1-like activation enzyme ATG7 in mice led to centralized nuclei in the glycolytic muscle, whereas, in a healthy state, nuclei should migrate to the periphery of muscle cells. However, only in long-term autophagy ablation were the twitch kinetics and force reduced and markers of apoptosis increased [125]. Importantly, in a mouse model of VCP-induced IBMPFD/ALS, the presence of insulin activates mTOR and inhibits autophagy, leading to a partial rescue of the phenotype, while rapamycin, through the inhibition of mTOR, activates autophagy, leading to more weakness, vacuolization and atrophy [126].

Inhibiting autophagy or mutant VCP appears to rescue cells and improve phenotypes, while the excessive activation of autophagy appears to be detrimental. The mechanism by which VCP variants cause cell death in muscle and neurons is unknown at this time and warrants further research. It is also not firmly established that the characteristic inclusion bodies actively contribute to cell death or if they are merely by-products of these dysfunctional systems.

## 5. Roles of VCP in the Nucleus

VCP plays many roles in the nuclear compartment as a mediator of DNA replication, DNA damage response systems and mitosis. However, there are few studies that examine the relationship between MSP and the nuclear role of VCP as a causative disease mechanism. It is plausible that poor genome integrity could impact both terminally differentiated tissues affected by MSP1 (such as neurons or muscle), or tissues with ongoing replication and remodeling, such as bone. Recently, a study in mice found that nuclei in mature muscle appeared to be able to undergo mitosis, challenging the longstanding dogma that myonuclei are post-mitotic [127]. Such a finding, should it be supported in future studies, may force us to reconsider potential pathogenic mechanisms in muscle disease. For instance, VCP’s role in genome integrity might impact nuclei division in mature muscle or it may contribute to the depletion of a viable pool of satellite cells necessary for the repletion of muscle cells or muscle nuclei [128]. There is some precedence for such a speculation, as abnormal satellite cell activity has been observed in a dystrophic (mdx) mouse model. Where normal muscle cells would enter quiescence, dystrophic muscles exhibit continued proliferation and differentiation [129]. There also appears to be significant changes in myogenic differentiation programming in facioscapulohumeral muscular dystrophy, indicating issues with satellite cells and myoblasts that are in the proliferative stages [130].

**Figure 3:**
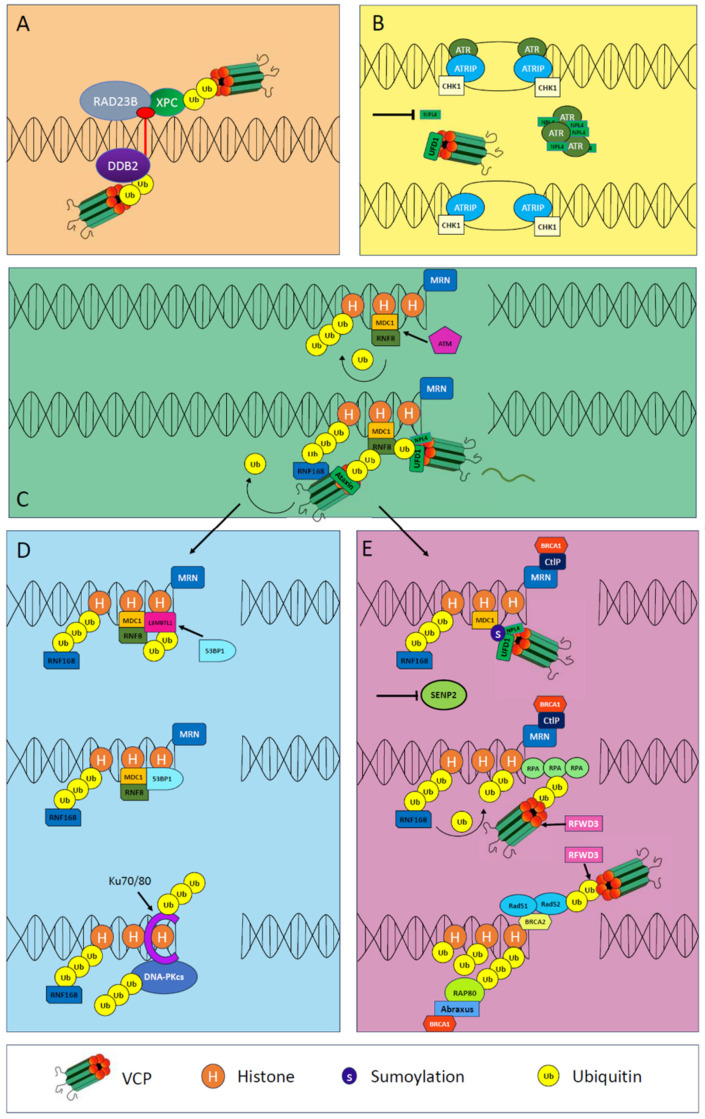
**Diverse functions of nuclear VCP in the DNA repair pathways.** (**A**) VCP in nucleotide excision repair, (**B**) VCP in ATR-mediated DNA damage response, (**C**) VCP in ATM-mediated DNA damage response, (**D**) VCP in non-homologous end joining and (**E**) VCP in homologous recombination.

Skeletal muscle and bone both contain multinucleated cells. If abnormalities in the differentiation or organization of multiple nuclei contribute in some way to disease, perhaps this is another link between the development of myopathy and Paget’s disease of Bone (PDB) in MSP. If the role of VCP in genome integrity does indeed at some point impact the differentiation of hematopoietic progenitors into osteoclasts or osteoclast fusion and satellite cell activity in skeletal muscle, perhaps VCP’s contribution to MSP is two-fold, as follows: dysfunction in autophagy and the DNA damage system cause considerable cellular stress, while at the same time impairing replenishment of nuclei in muscle or promoting abnormal fusion in osteoclasts. SQSTM1 is another key autophagy protein that induces MSP and has very similar functional features to VCP. Like VCP, it is shuttled between the cytosol and the nucleus and participates in DNA damage repair [131]. A discussion of the various genome integrity maintenance functions of VCP is provided and summarized in Figure 3.

### 5.1. DNA Replication and Mitosis

VCP has several roles in DNA replication as a major mediator of the ubiquitin and SUMO systems [132]. To initiate DNA replication, the many origins of DNA replication must be prepared and evenly distributed before the cell can transition from the G1 to the S-phase, termed origin licensing [128]. The ubiquitin system ensures that DNA does not initiate replication more than once; VCP extracts chromatin licensing, DNA replication factor 1 (CDT1) and origin recognition complex subunit 1 (ORC1) after DNA replication begins [132,133]. In order for DNA replication to terminate, VCP must extract the cell division cycle 45 (cdc45), Mcm2–7 helicase and GINS (CMG) replicative helicase from dsDNA [132,134]. VCP-UFD1L-NPLOC4 recognizes and extracts MCM7, a ubiquitinated subunit of the CMG helicase during replisome disassembly [132,135,136], with FAS-associated factor 1 (UBXN3/FAF1) being an additional participant after DNA damage [132,135].

### 5.2. VCP and the DNA Damage Response

Another potential function of VCP as it relates to MSP is the roles that VCP plays in maintaining genome stability. The SUMOylation of VCP permits the translocation of VCP to the nucleus, and many common *VCP* mutations, including those causing R155C, R159H, R95G, G97E and A232E, lead to the reduced SUMOylation of VCP [27]. The role of the DNA damage response pathway has been hypothesized as a common mechanism among polyglutamine disease, as VCP binds to polyglutamine disease proteins ataxin-1, ataxin-7, androgen receptor and huntingtin [137]. VCP and VCP adaptors have targets for cancer treatment, as inhibiting VCP induces DNA damage and kills some types of cancer cells [138]. Mutations at codon 470 and 616 in the D1-D2 linker region that are associated with ATPase hyperactivity are resistant to VCP inhibitors [139]. Phosphorylation of C-terminal serine 784 activates the VCP DNA damage response [140,141,142,143]. The recruitment of VCP to double stranded DNA breaks relies on the VCP cofactors UFD1-NPL4 [144]. When the VCP-UFD1-NPL4 complex is impaired, K48 ubiquitin chains accumulate at DNA damage sites, which suggests that the complex may play a role in extracting k48 ubiquitinated proteins [144]. VCP is involved in many DNA damage response pathways, including nucleotide excision repair, ATR- and ATM-mediated responses and homologous and non-homologous end-joining [145,146,147,148,149,150,151,152].

### 5.3. Nucleotide Excision Repair

When DNA is damaged by UV light or bulky base adducts, the cell responds to this by engaging the nucleotide excision repair DNA damage response pathway. Key proteins in the DNA damage response pathway include xeroderma pigmentosum C (XPC), which forms a complex with RAD23 homolog B and nucleotide excision repair protein (RAD23B) [153]. Together, they are essential for recognizing DNA damage in the early stages of the nucleotide excision repair pathway [153]. When damage recognition proteins stay localized to the DNA damage site for too long, the DNA damage excision repair machinery cannot function efficiently, causing an increase in DNA excision repair lesions [154]. VCP complexes participate in the removal of damage recognition proteins XPC and DDB2 [154], and the inhibition of VCP leads to an increase in ubiquitinated XPC at the site of the lesion, indicating that VCP is important in the removal of XPC [145].

### 5.4. ATR-Mediated DNA Damage Response

The ATR-mediated DNA damage response addresses both single-stranded and double-stranded DNA breaks [155]. Ataxia telangiectasia and Rad3-related (ATR) is a kinase that becomes activated in response to oxidative DNA damage, ionizing radiation and replication stress, activating downstream signal transduction to promote the repair of single-stranded and double-stranded breaks [156]. The VCP adaptor Npl4 has been used as a potential anti-cancer target in vitro [146]. Inhibiting Npl4 led to more DNA damage and increases in the number of active origins of replication, which indicate greater replication stress [146]. The ATR system responds to replication stress as a part of the ATR interacting protein–ataxia telangiectasia and Rad3-related–checkpoint kinase 1 (ATRIP-ATR-CHK1) complex [146]. The ATRIP-ATR-CHK1 complex prevents the collapse of ssDNA at replication forks, as, if ssDNA collapses, it can lead to double stranded breaks [146]. The authors suggest that the defects in the ATR-mediated pathway occur because the ATR kinase protein becomes trapped in Npl4 aggregates, rather than being due to defective segregase activity of VCP [146]. Spartan (DVC1/SPRTN) is a metalloprotein VCP adaptor that permits the recruitment of VCP to stalled replication forks [148]. When the SHP domain of DVC1 was mutated, DVC1 and VCP interaction was impaired [148].

### 5.5. ATM-Mediated DNA Damage Response: Homologous Recombination or Non-Homologous End-Joining?

The ATM-mediated DNA damage response is activated following double-stranded breaks and UV-induced lesions [157,158]. Several regulators upstream of HR and NHEJ determine the fate of the double-stranded break repair, including tumor suppressor p53-binding protein 1 (53BP1), mediator of DNA damage checkpoint protein 1 (MDC1), gamma H2A histone family member X (yH2AX), E3 ubiquitin–protein ligase RNF8 and RNF168 (RNF8/RNF168) and breast cancer type 1 susceptibility protein (BRCA1) [159].

RNF8 is recruited to broken chromosomes in the early stages of the DNA damage response and is an essential E3 ubiquitin ligase in the double-stranded DNA break response pathway [160]. Functionally, RNF8 works by adding ubiquitin to K48 and K63 residues on chromatin-associated proteins [157,158], which triggers the recruitment of the E3 ubiquitin ligase RNF168, which, in turn, activates other downstream proteins [144,160,161,162,163,164,165]. VCP regulates the amount of available RNF8 at the damage site and, if RNF8 becomes ubiquitinated, VCP targets and removes it from the damage site. However, VCP can also interact with the VCP cofactor ataxin-3 [150]. Ataxin-3 binds to the N-terminal domain of VCP, acting as a deubiquitinase (DUB), and can also remove ubiquitin from RNF8, preventing RNF8 from being removed and degraded prematurely [150]. Additionally, the ubiquitination of K48 and K63 residues by RNF8, promotes the activity of RNF168, which further promotes the addition of K63 ubiquitin chains to histone lysine residues [144,160,161,162,163,164,165]. Subsequently the VCP-UFD1-NPL4 complex is recruited to the DNA break, as the Npl4 factor can bind to the K63 chains via its zinc finger domain [151]. Here, VCP-UFD1-NPL4 promotes the release of L3MBTL histone methyllysine binding protein 1 (L3MBTL1) from chromatin, which allows for the recruitment of 53BP1 and activates the NHEJ pathway. The recruitment of BRCA1/breast cancer type 2 susceptibility protein (BRCA2)/DNA repair protein RAD51 homolog 1(Rad51), which behave as an antagonist to 53BP1, promote homologous recombination [151,159,166]. One study observed that, when VCP is knocked down, both 53BP1 and BRCA1 fail to accumulate at double-stranded DNA breaks, implying that VCP pays a role in both homologous and non-homologous repair [144].

### 5.6. Non-Homologous End-Joining DNA Damage Response

53BP1 is the upstream regulator of NHEJ and has two functions: limiting dsDNA resection and promoting oligomerization for double-stranded break synapsis [159]. When these molecular events occur primarily in the G1 phase, NHEJ is the preferred mechanism, but NHEJ can be active in other phases of the cell cycle [159]. Following the initiation of NHEJ, the Ku70/80 heterodimer senses and binds to double-stranded breaks to facilitate the recruitment of other NHEJ factors such as DNA-PKcs [152]. Ku70/80 must be modified with ubiquitin chains preceding its removal from the site of DNA damage, which is regulated by VCP [152]. VCP is also involved in regulating the degradation of DNA-PKcs via the proteasome [167]. When VCP is knocked down, Ku70/80 and DNA-PKcs accumulate at the DNA damage sites, impairing repair [152,167].

### 5.7. Homologous Recombination DNA Damage Response

Ionizing radiation and UV light cause double-stranded DNA breaks [148]. In response, the ATM-mediated DNA damage response initiates the phosphorylation of MDC1, which promotes MDC1 binding to histone proteins, followed by the recruitment of RNF8/RNF168 [149]. Sentrin-specific protease 2 (SENP2) is a deSUMOylase that removes SUMO groups from MDC1. When SUMO groups are removed, MDC1 is retained at the DNA damage site, and, for homologous recombination to proceed, MDC1 must be removed so that downstream proteins can bind. Thus, the prolonged retention of MDC1 at the DNA damage site promotes error-prone NHEJ and inhibits HR [149]. Inhibiting the activity of SENP2, on the other hand, results in SUMO groups building up on MDC1, which is then cleared from the DNA damage site by RNF4-VCP, thus impeding NHEJ and allowing HR to proceed [149].

VCP co-immunoprecipitates with replication protein A2 (RPA2) and RAD51, and, when VCP is depleted, ubiquitin accumulates on replication protein A1 (RPA1) and RPA2 [147]. RFWD3 ubiquitinates RPA1 and RAD51, followed by the removal of RPA1 and RAD51 by VCP and their degradation by the proteasome [147]. The removal of RPA1 and RAD51 facilitates the recruitment of late homologous recombination factors [147]. The VCP cofactor Ufd1 facilitates the interaction between VCP and SUMOylated proteins [168]. VCP associates with SUMOylated Rad52 while Rad52 is a part of the Rad52-Rad51 complex, removing the interaction between Rad52-Rad51 and preventing their association with DNA.

Given VCP’s extensive role across DNA damage response systems, including nucleotide excision repair, ATM- and ATR-mediated DNA-damage response and homologous and non-homologous end-joining, it may be worthwhile to investigate how VCP variants impact nuclei in MSP1. Although muscle undergoes little cellular turnover, a decrease in the efficacy of the DNA damage response system, in addition to the autophagy system, could potentially contribute to the pathology of MSP1. Nuclei with more DNA damage are more likely to express somatic mutations, producing ineffective essential proteins, contributing to the pool of cellular waste that must be cleared by autophagy. It is also possible that myonuclei that are compromised, or that are not efficiently replaced by satellite cells during cellular turnover, could lead to a reduction in muscle fiber size and growth potential, especially during aging. In 2020, a study by Cramer et al. demonstrated that, when satellite cell fusion was blocked in neonatal mice, myofibers sizes were reduced, while nuclei were forced to increase mRNA concentrations to compensate for fewer total nuclei [169]. Since VCP plays such a profound role in genome stability, perhaps pathogenic VCP causes a reduction in healthy nuclei, contributing to myopathy by reducing fiber size.

## 6. Conclusions

Although the major roles of VCP in protein quality control are presumed to be the major mechanisms implicated in MSP, the incredible functional diversity and pleiotropic effects of VCP also imply that other mechanisms may be relevant and require further study. VCP cooperates with the 26S proteasome, the main pathway for protein degradation, to manage the protein quality control system. In the nucleus, VCP regulates cell cycle control and the DNA damage response by coordinating proteins at DNA damage sites. In the cytosol, VCP regulates responses to cellular stress by forming and clearing stress granules, facilitating ERAD, autophagy, mitophagy and lysophagy, and VCP may also be involved in apoptosis. The complexity of VCP’s diverse molecular functions is also mirrored by the variability in clinical dysfunction caused by pathogenic variants in *VCP*. The relationship between specific molecular functions of VCP and the spectrum of clinical presentations remains poorly understood, and, in general, genotype–phenotype correlation is still difficult to demonstrate. Certainly, VCP plays many yet-to-be-identified roles in different cellular systems. Given that the role of VCP extends to so many cellular systems, it makes it difficult to ascertain which dysfunction leads to which clinical phenotype. The majority of MSP cases are related to variants at positions 155 and 159, but the phenotypic variability is extensive, suggesting that other genetic or epigenetic factors and/or environmental factors may interact. To better narrow down a causative mechanism in a given tissue, we advise that, when possible, experiments should include one or two other MSP genes such as *SQSTM1* or *HNRNPA2B1*, as this may help identify common mechanisms of dysfunction in MSP. Studies of large cohorts of patients who have common variants in VCP may allow for the identification of genetic modifiers or other factors that contribute to phenotypic variability. Even though pathogenic variants in *VCP* typically lead to multisystem disease, in general, the affected systems predictably include certain tissue types (primarily skeletal muscle, the cerebrum, motor neurons and osteoclasts). Even though VCP is ubiquitously expressed and participates in numerous crucial cellular functions, pan-systemic disease is not observed. There are likely to be important reasons for this differential tissue vulnerability. For central and peripheral nervous system tissues, which have large and long-lived terminally differentiated cells, it is likely that cellular damage accumulates over time due to the dysfunction of VCP’s myriad roles. Two of the affected tissues (skeletal muscle and osteoclasts) are multinucleated cells, implying that there may be increased proteostatic needs for these cell types. However, cardiac muscle contains cells that are both multinucleated and a long-lived differentiated cell type. Despite this, cardiac disease due to VCP variants is described only in limited series or cases. Cardiac muscle may have reduced vulnerability compared to skeletal muscle because a large fraction of cardiomyocytes are mononuclear [170], therefore may have relatively lower proteostatic needs. Involvement in other tissues may also occur but could be subclinical or simply absent for cell types that are shorter lived and rapidly replaced by progenitor cell populations.

The tissue specificity of VCP-MSP, and the precise mechanisms that result in disease, have important therapeutic implications. Studies are currently in progress to identify future therapies [171]. A better understanding of the most relevant mechanisms to disease would allow for the development of targeted treatments focused on these mechanisms and directed to specific tissue types that are relevant to the common phenotypes.
